# Circulating Biomarkers to Predict Diabetic Retinopathy in Patients with Diabetic Kidney Disease

**DOI:** 10.3390/vision7020034

**Published:** 2023-04-09

**Authors:** Jonny, Laurencia Violetta, Arief S. Kartasasmita, Rudi Supriyadi, Coriejati Rita

**Affiliations:** 1 Nephrology Division, Department of Internal Medicine, Gatot Soebroto Indonesia Army Central Hospital, Jakarta 10410, Indonesia; 2 Faculty of Medicine, Universitas Padjajaran, Bandung 40132, Indonesia

**Keywords:** biomarkers, diabetic retinopathy, diabetic kidney disease, microangiopathy, diabetes mellitus

## Abstract

The purpose of this review is to outline the currently available circulating biomarkers to predict diabetic retinopathy (DR) in patients with diabetic kidney disease (DKD). Studies have extensively reported the association between DR and DKD, suggesting the presence of common pathways of microangiopathy. The presence of other ocular complications including diabetic cataracts may hinder the detection of retinopathy, which may affect the visual outcome after surgery. Unlike DKD screening, the detection of DR requires complex, costly machines and trained technicians. Recognizing potential biological markers related to glycation and oxidative stress, inflammation and endothelial dysfunction, basement membrane thickening, angiogenesis, and thrombosis as well as novel molecular markers involved in the microangiopathy process may be useful as predictors of retinopathy and identify those at risk of DR progression, especially in cases where retinal visualization becomes a clinical challenge. Further investigations could assist in deciding which biomarkers possess the highest predictive power to predict retinopathy in clinical settings.

## 1. Introduction

Diabetes mellitus (DM) leads to microvascular complications affecting the eyes, nerves, and kidneys, significantly affecting the patients’ quality of life [1]. Approximately half of chronic kidney disease cases are accounted for by diabetic kidney disease (DKD) [2], while almost all type 1 and over 50% of type 2 diabetic patients will suffer from diabetic retinopathy (DR) [3]. About 45–70% of DKD patients on dialysis present with retinopathy of any stage, and the presence of DR is associated with increased overall mortality risk [4].

Compared to DKD screening, which can be easily performed by simple urinalysis, DR screening requires annual ophthalmology visits separately from routine diabetes consultations, often leading to poorer compliance. Furthermore, the presence of other ocular complications including diabetic cataracts may hinder retinopathy detection, especially in severe cases in which posterior segment visualization becomes a challenge [5]. Extraction is the standard management for cataracts, however, the exacerbation of pre-existing DR and increased long-term risk of developing new retinopathy have previously been described [6,7]. Routine dialysis for DKD also aggravates the already malfunctioned retinal capillaries leading to diabetic macular edema (DME) [8]. Thus, comprehensive ophthalmological screening is needed to identify DR and provide the appropriate management. As technologies utilized in retinal screening are costly and not universally available, it raises the interest in discovering biomarkers that can accurately predict the presence of retinopathy and aid in the development of effective therapies.

We performed a non-systematical narrative review of currently available circulating biomarkers associated with the common pathways involved in the pathogenesis of DKD and DR. We identified articles relating to biomarkers of retinopathy, particularly those that exhibit promising roles as clinical markers in the screening and evaluation of retinopathy in diabetic patients with kidney dysfunction, through Internet searches in Google Scholars and PubMed using the keywords: biomarkers, diabetic retinopathy, and diabetic kidney disease. We only included studies relevant to blood and urinary biomarkers, while those of vitreous biomarkers were excluded.

## 2. Association between Diabetic Retinopathy and Diabetic Kidney Disease

**Figure 1:**
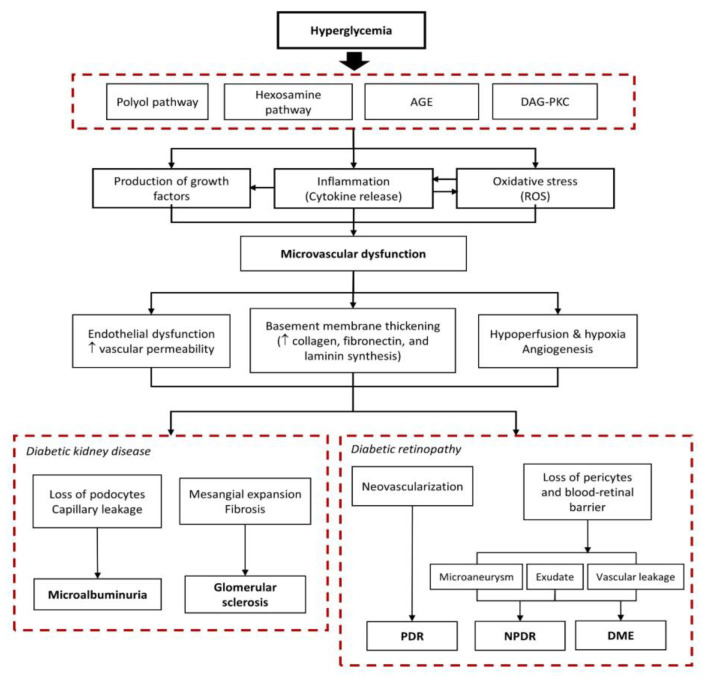
Hyperglycemia-induced biochemical pathways in the development of diabetic retinopathy and diabetic kidney disease. AGE, advanced glycation end-products; DAG-PKC, diacylglycerol-protein kinase C; ROS, reactive oxygen species; PDR, proliferative diabetic retinopathy; NPDR, non-proliferative diabetic retinopathy; DME, diabetic macular edema.

Studies have suggested multiple hyperglycemia-induced biochemical pathways resulting in dysfunction and the loss of endothelial cells, retinal pericytes, and renal podocytes [9,10]. DKD and DR begin with a chain of cellular mechanisms that activate the polyol, hexosamine, and diacylglycerol (DAG)-protein kinase C (PKC) pathways, non-enzymatic glycation, and mitochondrial stress, leading to the enhanced production of advanced glycation end-products (AGEs), reactive oxygen species (ROS), and various inflammatory cytokines and pro-angiogenic growth factors (Figure 1).

Researchers have reported that one microvascular complication can predict the existence of other complications, in which DR was proven to be a key risk factor for developing DKD, and vice versa. Patients with DR were 10 times at greater risk of acquiring renal dysfunction, where DKD occurred in approximately 19.2% of patients with non-proliferative DR (NPDR) and up to 41.2% in those with proliferative DR (PDR) [11]. DR was described as an important predictor of microalbuminuria progression and its severity was related to a drop in the estimated glomerular filtration rate (eGFR) and a rise in the serum creatinine (Scr) levels [12]. Even in normoalbuminuric patients, the presence of DR worsened the urine albumin excretion rate (UAER) and doubled the rate of progression to microalbuminuria [13]. In type 1 DM (T1DM) patients with DKD, high blood urea nitrogen (BUN) and Scr levels were important predictors of retinopathy, in which baseline gross proteinuria increased the risk of developing PDR by 12.5% annually [14]. Likewise, in type 2 DM (T2DM) patients, eGFR, albuminuria, and UAER were strongly associated with DR incidence, and high UAER can be found predominantly in patients with severe NPDR and PDR [15].

## 3. Current Technologies to Detect Retinopathy

The detection of DR was widely conducted by direct ophthalmoscopy to find the presence of clinical signs of non-proliferative and proliferative changes [3]. The standard seven-field fundus photography provides high-quality retinal images and has become the gold standard for DR diagnosis and staging [16]. With the advancements in fundus cameras, recent studies have emphasized the superiority of non-mydriatic fundus photographs compared to ophthalmoscopy with comparable diagnostic accuracy to mydriatic photography [17]. Further developments include the creation of an automated algorithm with over 90% in sensitivity and specificity in detecting DR and DME, and the introduction of ultra-widefield (UWF) color photography that allows up to a 200° visualization of the retina, providing identification of peripheral retinal pathologies [18,19]. Optical coherence tomography (OCT) offers a detailed cross-sectional structure of the retina and choroid and was more sensitive than fundus photography in detecting and distinguishing different DME subtypes [17]. OCT angiography (OCTA) and fundus fluorescein angiography (FFA) were developed to visualize any microaneurysms, neovascularizations, and regions of non-perfusion to assist in DR staging [20]. Nevertheless, all of the aforementioned methods are costly and require specialized equipment, trained technicians, and experts to interpret these digital images, which may not be widely available to the general population. Moreover, retinal photography does not detect other diabetic ocular complications including cataracts or glaucoma.

## 4. Biomarkers to Predict Retinopathy in Patients with Diabetic Kidney Disease

Several clinical markers of DR and DKD have been studied extensively to assist in detecting early disease, identify patients at risk for progression, and developing new treatments. Glycated hemoglobin (HbA1c) is the most routinely measured clinical biomarker as glycemic control is a recognized risk factor for DKD and DR. Retinopathy and microalbuminuria were associated with HbA1c >7.0% with increased risk of sight-threatening DR and macroalbuminuria at a HbA1c level exceeding 7.5–8.0%. Microalbuminuria was more specific in predicting DR progression than eGFR, where microalbuminuric patients with eGFR <60 mL/min had a higher risk of DR deterioration compared to those with similar eGFR, but normoalbuminuric [21]. Moreover, the urine albumin–creatinine ratio (UACR) was reported to be a better predictor of DR than eGFR. In patients with T2DM, eGFR <60 mL/min and UACR >30 mg/g were associated with an increased risk of developing PDR and DME [22].

Aside from these traditional biomarkers, various biomarkers of DR have emerged to aid in predicting DR incidence and progression including those associated with the common pathways involved in DR and DKD development (i.e., hypoxia, oxidative stress, inflammation, endothelial dysfunction, and angiogenesis) as well as a new category of molecular biomarkers. As opposed to vitreous markers of retinopathy, here, we discuss the systemic markers that can be obtained easily such as blood and urinary markers (Table 1).

### 4.1. Biomarkers Related to Glycation and Oxidative Stress

Hyperglycemia and oxidative stress accelerate the non-enzymatic glycation reaction, a key mechanism contributing to the release and accumulation of AGEs in renal and retinal tissues. AGE interaction with their receptors (RAGE) exacerbates the production of ROS, pro-angiogenic factors, and cytokines [9,10]. Among the AGEs, the concentration of serum N-epsilon-carboxy methyl lysine (CML) and pentosidine markedly increased in both DR and DKD, especially in subjects with PDR compared to mild or severe NPDR [23]. In the EUROCONDOR trial, serum CML concentration was linked to retinal thickness and neurodysfunction, allowing for the identification of early abnormal DR findings and monitoring of its progression [24]. Elevated blood levels of RAGEs in T2DM patients with PDR compared to subjects with NPDR or without DR have also been described [25]. Dyslipidemia is a well-known risk factor of DR due to its effect on free radicals, ROS, and pro-inflammatory molecule production [26]. Apoliproteins (Apo), suggested to be a better estimate of lipid levels compared to conventional high-density (HDL) and low-density lipoproteins (LDL), consist of several subclasses including the extensively studied Apo-A and Apo-B. While Apo-A1, as a component of the HDL protein, possesses anti-oxidant and anti-inflammatory properties, Apo-B is the main apolipoprotein of LDL, and in contrast, exerts atherogenic effects in the retinal vessels [26]. Elevated Apo-A1 and low Apo-B levels have been shown to be protective factors of DR, and an increased Apo-B/Apo-A1 ratio was associated with a more severe form of DR [27]. Zhang et al. [28]. showed that a serum Apo-A1 level ≥7.4 umol/L was associated with a reduced risk of having DR and its progression to NPDR and PDR by 15% and 35%, respectively. Another study also revealed the circulating AGE-based modification of low-density lipoprotein (AGE-LDL) and oxidized LDL (Apo-B100) as independent predictors for DR progression, as increased expressions was related to apoptosis of the retinal capillary pericytes [29].

Asymmetric dimethylarginine (ADMA), symmetric dimethylarginine (SDMA), and I-arginine are involved in the nitric oxide pathway and reflect the state of oxidative stress, all of which increased significantly in severe NPDR, PDR, and DME [30]. Another identified marker of oxidative damage is 8-hydroxy-2′-deoxyguanosine (8-OHdG), concentrations of which were associated with HbA1c. A high level of urinary 8-OHdG was found in T2DM patients with PDR compared to those with NPDR or without DR [31]. Myeloperoxidase (MPO) is a pro-oxidative enzyme that contributes to the production of AGEs and ROS and high levels were positively correlated to UACR and DKD progression [32]. Sinha et al. [33]. reported significantly higher serum anti-MPO antibody levels in PDR and NPDR patients compared to the controls (37.27 RU/mL, 21.51 RU/mL, and 16.94 IU/mL, respectively), with a strong correlation to a decrease in visual acuity.

Anti-oxidant thiols including reduced glutathione (GSH) and protein-bound thiols play a role in diminishing the highly reactive superoxide radicals in microangiopathy, where increased incidence and progression of retinopathy were linked to the low serum concentration of thiols in diabetic patients [34]. A recently identified biomarker of early DR is S100A12 or calgranulin C, a calcium-binding pro-inflammatory protein that acts as a receptor for AGE binding protein. The plasma S100A12 level was independently associated with DR onset and progression in T2DM patients, with a diagnostic sensitivity and specificity of 78.1% and 77.0%, respectively [35].

### 4.2. Biomarkers Related to Inflammation and Endothelial Dysfunction

Interleukins (ILs) and tumor necrosis factor-α (TNF-α) are pro-inflammatory molecules whose circulating levels have been shown to be elevated in diabetes [9]. High systemic concentrations of IL-1β, IL-6, and IL-8 were associated with the presence of DR [36] and DKD [37] and positively correlated to disease severity. In particular, plasma IL-6 could potentially be used to assess the progression of NPDR to PDR and the exacerbation of interstitial inflammation in DKD [36]. Furthermore, these cytokines also upregulate the production of vascular endothelial growth factor (VEGF), which was associated with advanced DR, and can be used as potential biomarkers for treatment evaluation with anti-VEGF therapy. In the Wisconsin Epidemiologic Study of Diabetic Retinopathy, plasma TNF-α was found to be increased along with DR severity in patients with DKD [38]. Kuo et al. [39]. demonstrated that in T1DM patients with renal dysfunction, those with PDR displayed higher levels of serum soluble TNF receptors 1 (sTNF-R1) and 2 (sTNF-R2) compared to those without DR. Since these receptors modulate the biological activity of TNF-α, sTNFRs could be more sensitive in assessing DR severity.

Intercellular adhesion molecule-1 (ICAM1) and vascular adhesion molecule-1 (VCAM1) are upregulated in response to inflammatory cytokines [36]. Circulating levels of ICAM1 and VCAM1 gradually rise along with each step of DR advancement from NPDR to PDR [36], and high levels led to increased risk of retinopathy in patients with co-existent DKD [38]. Other adhesion molecules, E-selectin, P-selectin, and monocyte chemoattractant protein-1 (MCP1), were also upregulated, and elevated serum levels were correlated to severe retinopathy and renal damage [37]. The chemokines regulated on activation, normal T-cell expression, and presumably, secreted (RANTES)/CCL5 was also overexpressed in diabetes, contributing to inflammation within glomerular and tubular cells and exhibiting a strong association with DR progression [36]. 

Li et al. [40] reported a relationship between the serum and urinary levels of mannose-binding lectin (MBL), a lectin pathway associated with the activation of various complement cascades including C1q, C4d, C3a, and C5a, the levels of which were positively correlated with Scr, proteinuria, eGFR, and glomerular lesions in DKD patients. In another study, serum MBL was an independent risk factor for DR and the development of sight-threatening DR in T2DM patients [41]. Meanwhile, the classical pathway of the complement system lead to the formation of autoantibodies that damage retinal pericytes in the early stage of DR, as evidenced by the high plasma levels of complement components such as C3a and C5a in DR compared to non-DR patients [42]. 

Hyperhomocysteinemia has been implicated in the inflammation process through leukocyte recruitment and the production of pro-inflammatory cytokines, specifically IL-8 and MCP-1. Elevated plasma homocysteine (Hcy) was linked to impaired renal function, development of albuminuria, and increasing severity of DKD [43]. Klein et al. [38] reported an association between high Hcy level with a greater risk of developing DR and progression to PDR and DME in patients with renal dysfunction. A similar finding was demonstrated in a trial involving 175 T2DM patients, where the highest level of Hcy was observed in the PDR group (18.2 umol/L ± 5.6) compared to the NPDR (7.8 umol/L ± 6.4) or non-DR (12.1 umol/L ± 6.8) groups [44]. Retinol-binding protein 4 (RBP4) is an established biomarker of renal damage associated with various inflammatory factors, where its serum and urinary levels were negatively correlated to eGFR, independent of the presence of albuminuria [45]. A significantly elevated RBP4 in T1DM patients with DR was described by Li et al. [46], with a 5% and 9% increased risk of DR development and progression to sight-threatening DR for every 1 ug/mL rise in the plasma RBP4 level, respectively.

C-reactive protein (CRP) is a commonly used biomarker of systemic inflammation, which relates strongly to the incidence and progression of DKD and DR. In a meta-analysis involving 3679 T1DM and T2DM patients, Song et al. [47]. discovered higher CRP levels in patients with DR than non-DR, particularly in PDR compared to NPDR. Diabetic patients with macro- and micro-albuminuria also exhibited high circulating CRP than those without albuminuria [48]. Serum amyloid A (SAA) is another systemic inflammatory marker that has been shown to markedly increase in micro- and macro-albuminuric patients [37] and diabetic retinopathy [49]. In DKD patients, elevated circulating Pentraxin 3 (PTX3), an acute-phase reactant that reflects endothelial dysfunction, may be used as a biomarker in detecting early renal damage [50]. Yang et al. [51] observed a greater proportion of severe retinal complications along with the plasma level of PTX3 >1150 pg/mL and may be more accurate in predicting DR and its development compared to CRP (sensitivity, 53.3% vs. 51.1%; specificity, 91.7% vs. 70.8%, respectively).

Adiponectin (APN) plays a role in anti-inflammation via the inhibition of TNF-α, VCAM-1, and ICAM-1, and a low level has been associated with increased diabetes risk [52]. Omae et al. [53] suggested the protective role of APN in T2DM patients with early DR, and high plasma adiponectin was linked to improved retinal microcirculation. Serum level of a novel adipokine biomarker, C1q complement/TNF-related proteins-3 (CTRP3), was also shown to be markedly reduced in T2DM patients with DR and associated with progression to PDR [54]. Adropin is another inhibitor of inflammation that was significantly decreased in the serum and vitreous of patients with PDR than those with NPDR [55]. Likewise, lipoxin A4 (LXA4) exhibits protective properties against inflammation, and low serum LXA4 was observed in patients with both NPDR and PDR [56]. The identification of circulating APN, CTRP3, adropin, and LXA4 may also provide insights on the therapeutic effect of an anti-inflammatory as potential management of DR. 

### 4.3. Biomarkers of Basement Membrane Thickening

Microangiopathy creates an imbalance in the rate of basement membrane (BM) degradation and synthesis, leading to the overexpression of various BM components [9,10]. Laminin-P1 is a pepsin-resistant fragment of laminin that is synthesized by endothelial, pericytes, and mesangial cells. Previous studies have suggested the use of circulating laminin-P1 as an early marker for DR presence and severity [57]. Evidence from the EUROCONDOR showed that the baseline serum laminin-P1 was correlated to the increase in the ganglion cell layer-inner plexiform layer and overall retinal thickness [24]. Moreover, laminin-P1 was reduced in patients who were given somatostatin, indicating its usefulness in monitoring the therapeutic effectiveness of early DR management. Collagen type IV is a main BM matrix protein, and elevated urinary and serum levels were correlated with DR and DKD. The high serum concentration of collagen IV corresponds to an increased level of vitreous collagen IV that was associated with Hba1c and the severity of DR [58].

Metalloproteinases (MMPs) are key enzymes responsible for the degradation of extracellular matrix proteins, which are modulated by tissue inhibitors of metalloproteinases (TIMPs). A disequilibrium of the ratio of MMPs to TIMPs occurred in DM, which led to DR. In particular, increased levels of systemic MMP-9 and high MMP-9/TIMP-1 ratio have been reported in T1DM patients with DR compared to those without DR [59]. A similar finding was observed in another study with T2DM subjects, in which those with PDR exhibited high levels of circulating MMP-2 and -9 [60]. In the EURODIAB trial, high plasma levels of MMP-2, -3, and -10 were found to be associated with severe albuminuria, however, only MMP-2 was correlated to the development and severity of retinopathy [61].

### 4.4. Biomarkers of Angiogenesis and Thrombosis

A positive relation between circulating VEGF concentration and DR development and progression has previously been demonstrated [62], as suggested by the role of VEGF in neovascularization. The accuracy of serum VEGF had over 90% sensitivity and specificity in predicting the presence of DR and discriminating between the different stages of DR [63]. In DKD patients on dialysis, it was suggested that that erythropoietin (EPO) administration was a risk factor of DR deterioration in this population. EPO is a systemic pro-angiogenic hormone that has been proven to contribute to the proliferative phase of DR. Serum EPO was shown to be positively correlated with the clinical stage of DR, with the highest concentration found in the PDR (9.95 mIU/mL) group compared to those of the with NPDR (7.0 mIU/mL) or without DR (6.9 mIU/mL) groups [64].

Low serum insulin-like growth factor-1 (IGF-1) reflects an increased activity of IGF-1 in tissues implicated in angiogenesis, inflammation, and endothelial injury, leading to DR and DKD [65]. The serum level of IGF-1 was negatively associated with DR progression, independently of HbA1c and diabetes duration [66]. Fibroblast growth factor-21 (FGF-21) is upregulated in DR as a compensatory response to endothelial dysfunction that aims to repair retinal microvascular lesions. In a cross-sectional study of 142 T2DM patients, the serum FGF-21 concentrations were markedly elevated in the DR group (103.5 pg/mL) compared to those without DR (99 pg/mL), although no association was found regarding the severity of DR as the FGF21 levels were comparable between the NPDR and PDR groups [67].

Elevated urinary TGF-β can be observed in the presence of microalbuminuria, while elevated circulating TGF-β was strongly related to the incidence of DR, especially PDR. In particular, serum TGF-β1 had 72% and 88% sensitivity and specificity to predict the occurrence of DR, respectively [68]. Hypoxia-inducible factors (HIF) is a key transcription factor that activates various genes of pro-angiogenic growth factors as an adaptive response to hypoxia. In T2DM patients with DKD, serum HIF-1α was positively correlated with Scr and BUN and negatively associated with eGFR [65]. A significant increase in serum HIF-1α was also seen in DR, particularly in those with grades 3 and 4 compared to lower DR grades [69]. Cystatin C is a well-established marker of kidney function that has been suggested to play a role in angiogenesis. Wong et al. [70] reported an association between a high level of cystatin C (≥1.12 nmg/L) in patients with DKD with approximately 14 times greater risk of developing severe NPDR and PDR.

Pigment epithelium-derived factor (PEDF) is a potent antagonist of VEGF. Reduced circulating PEDF levels have been demonstrated in diabetic patients with retinopathy and high urinary PEDF was independently correlated to microalbuminuria [71]. Calcitriol or 25-hydroxyvitamin D3 [25(OH)VD3] is another potent anti-angiogenic factor that may halt neovascularization through VEGF inhibition, and its level was negatively correlated with UACR, Scr, and BUN in T2DM patients with DKD [65]. A meta-analysis showed that diabetic patients with serum vitamin D levels <30 nmol/L had a 60% greater risk of developing PDR compared to those with vitamin D >75 nmol/L, suggesting the potential benefits of vitamin D supplementation to delay PDR progression [72].

Polat et al. [73] showed the elevation of various pro-thrombotic factors including the serum α2 antiplasmin, fibrinogen, plasminogen, and plasminogen activator inhibitor (PAI)-1 levels in early NPDR, which gradually increased along with the severity of DR. Results from the Veterans Affairs Diabetes Trial showed that PAI-1 was an independent risk factor for DR incidence in T2DM patients, specifically, a 12% greater risk of developing DR for each 10 ng/dL rise in the baseline serum PAI-1 level [74].

### 4.5. Other Novel Molecular Biomarkers

There has been growing interest in exploring epigenetics mechanisms of mRNA, microRNA (miRNA), long non-coding RNA (lncRNA), DNA methylation, and histone modifications, as they regulate various biological pathways in the pathophysiology of diabetic complications. The association between circulating mRNA encoding the retina-specific pigment-protein rhodopsin (RHO) was demonstrated by Hamaoui et al. [75], who reported a rising trend of RHO mRNA along with DR progression with the highest level observed in the PDR group. In a further study, Butt et al. [76] detected a reduction in circulating mRNA for retinal amine oxidase (RAO) and an increase in the RHO/RAO mRNA ratio as DR progressed. Further investigation showed that this ratio was better than RHO mRNA alone at differentiating mild or no DR from severe NPDR and PDR. Other identified potential circulating mRNAs include circulating retinal pigment epithelium-65 (RPE65) mRNA, whose concentrations rose with DR severity and retinoschisin mRNA, which in contrast, was reduced as DR progressed [77].

Meanwhile, miRNAs are short-coding RNAs that regulate various target genes including VEGF and fibronectin. In a study of T1DM patients, DR patients exhibited the overexpression of miR-221, although no statistical difference was found among different degrees of DR [78]. In another study by Liu et al. [79] on T2DM patients, the serum miR-221 was shown to be progressively upregulated as the DR severity increased and possessed the highest diagnostic efficiency compared to angiotensin II or VEGF. Zhou et al. [80] demonstrated significantly higher plasma miR-93 levels in DR subjects with a cutoff-value of 1.31 to detect the presence of DR with the diagnostic sensitivity and specificity of 73.3% and 89.24%, respectively. Other identified circulating miRNAs that were positively associated with DR progression include miR-21, miR-15a, miR-155, and miR-122. In contrast, several serum miRNAs such as miR-17-3p, miR-126, miR-423, miR-200b, and miR-20b were demonstrated to progressively decrease as PDR developed [81].

Long-coding RNAs (lncRNA) are novel molecular biomarkers that modulate both transcription and post-transcriptions of various target genes. Shaker et al. [82]. observed significantly increased levels of serum homebox antisense intergenic RNA (HOTAIR) and metastasis-associated lung adenocarcinoma transcript 1 (MALAT1) in T2DM patients with PDR compared to NPDR. In contrast, Toraih et al. [83] found a reduced expression of serum lncRNA MALAT1 and retinal non-coding RNA2 (RNCR2) in DR patients compared to subjects without DR, although no association was observed with the severity of DR. Furthermore, Zha et al. [84] discovered a downregulation of the long intergenic non-protein coding RNA p53 induced transcript (LINC-PINT) in patients with retinopathy, suggesting the potential role of LINC-PINT in halting DR progression. In addition, the association of hypermethylation of global DNA, 5,10-methylenetetrahydrofolate reductase (MTHFR), and miR-9-3 gene promoter with DR presence and severity have also been previously demonstrated [81].

## 5. Limitations and Future Directions

Research into the biomarkers presented in this review provides insights into the interconnecting complex pathomechanisms between diabetic microvascular complications. Identifying circulating biomarkers could be a useful additional tool in identifying DR in diabetic patients at any stage and predicting its risk of progression, especially in DKD patients with concomitant ocular complications where visualization of the posterior segment is difficult to achieve. Additionally, biomarkers relevant to pathways in diabetic complications are important contributors to the development of novel retinopathy treatment. The use of biomarkers may guide the need and choice of retinopathy treatment as well as monitor their therapeutic effectiveness.

Nonetheless, several limitations should be considered. First, as a systemic disease, complications of diabetes share mutual pathways, which presents a major challenge in the use of biomarkers. As a result, some biomarkers such as those related to non-enzymatic glycation, oxidative stress, inflammation, etc., may overlap and have a triggering role in other ocular diabetic complications including diabetic cataracts and diabetic glaucoma. Furthermore, many traditional risk factors including glycemic control, diabetes duration, genetic factors, smoking, hypertension, and dietary or lifestyle habit may influence the incidence and severity of retinopathy. Hence, combining multiple biomarkers rather than individual biomarkers with other clinical diagnostic tools may be useful in identifying the ocular pathology of diabetic patients. Moreover, despite being more readily accessible, systemic blood and urinary markers of retinopathy may not reflect the true state of retinopathy compared to intraocular biomarkers. For example, vitreous concentrations of interleukins and VEGF were strongly implicated with the severity and progression of DR, but not with the plasma levels, suggesting a stronger local over systemic process in severe retinopathy and the different degree of expression of some of the biomarkers in the blood versus retina [85,86].

## 6. Conclusions

Finally, the usefulness of these biomarkers in different populations such as among type 1 and type 2 diabetic patients and between individuals at different ages, ethnicities, and stages of retinopathy and kidney dysfunction still needs to be validated. Although promising, the clinical feasibility of these biomarkers is still lacking and requires further large-scale investigations and validation to address which specific or combination of biomarkers possess the highest predictive power to be used as a screening tool in routine clinical practice.

## Figures and Tables

**Table 1:** Potential biomarkers to predict diabetic retinopathy in patients with diabetic kidney disease.

Categories	Biomarkers	References
Biomarkers related to glycation and oxidative stress	Total Advanced glycation end-products (AGEs)	[9,10]
	N-epsilon-carboxy methyl lysine (CML)	[23,24]
	Pentosidine	[23]
	Soluble AGE receptors (sRAGE)	[25]
	Apolipoprotein (Apo)-A1, Apo-B	[26,27,28]
	AGE-low density lipoprotein (AGE-LDL)	[29]
	Oxidized LDL (Apo-B100)	[29]
	Reactive oxygen species (ROS)	[9,10]
	Asymmetric and symmetric dimethylarginine (ADMA, SDMA)	[30]
	I-arginine	[30]
	8-hydroxy-2′-deoxyguanosine (8-OHdG)	[31]
	Anti-myeloperoxidase (MPO) antibody	[32,33]
	Thiols, reduced glutathione (GSH)	[34]
	S100A12 protein (Calgranulin C)	[35]
Biomarkers related to inflammation and endothelial dysfunction	Interleukins (IL-1β, IL-6, IL-8)	[36,37]
	Chemokine C-C Motif Ligand 5 (RANTES/CCL5)	[36]
	E-selectin, P-selectin	[37]
	Monocyte chemoattractant protein (MCP)-1	[37]
	Tumor necrosis factor-α (TNF-α)	[38]
	Intracellular adhesion molecule (ICAM)-1	[36,38]
	Vascular adhesion molecule (VCAM)-1	[36,38]
	Soluble TNF receptors (sTNF-R1, sTNF-R2)	[39]
	Mannose-binding lectin (MBL)	[40,41]
	Complement components (C1q, C3a, C4d, C5a)	[40,42]
	Homocysteine	[38,43,44]
	Retinol binding protein 4 (RBP4)	[45,46]
	C-reactive protein (CRP)	[47,48]
	Serum amyloid A (SAA)	[49]
	Pentraxin (PTX)-3	[50,51]
	Adiponectin (APN)	[52,53]
	C1q complement/TNF-related proteins (CTRP)-3	[54]
	Adropin, Lipoxin A4 (LXA4)	[55,56]
Biomarkers of basement membrane thickening	Laminin-P1	[24,57]
	Collagen type IV	[58]
	Metalloproteinases (MMPs)	[59,60,61]
Biomarkers related to angiogenesis and thrombosis	Vascular endothelial growth factor (VEGF)	[62,63]
	Erythropoietin (EPO)	[64]
	Insulin-like growth factor (IGF)-1	[65,66]
	Fibroblast growth factor (FGF)-21	[67]
	Transforming growth factor-β (TGF-β)	[68]
	Hypoxia inducible factor (HIF)-1α	[65,69]
	Cystatin C	[70]
	Pigment epithelium derived factor (PEDF)	[71]
	25-hydroxyvitamin D3 [25(OH)VD3]	[65,72]
	Serum α2 antiplasmin	[73]
	Fibrinogen, plasminogen	[73]
	Plasminogen activator inhibitor (PAI)-1	[73,74]
Novel molecular biomarkers	Messenger RNA (mRNAs)—Rhodopsin (RHO), Retinal amine oxidase (RAO), Retinal pigment epithelium-specific 65 (RPE65), Retinoschisin	[75,76,77]
	MicroRNA (miRNAs)—miR-221, miR-93, miR-21, miR-15a, miR-122, miR-126, miR-17-3-p, miR-423, miR-200b, miR-20b	[78,79,80,81]
	Long-coding RNA (lncRNAs)—homebox antisense intergenic RNA (HOTAIR), metastasis-associated lung adenocarcinoma transcript (MALAT)-1, retinal non-coding RNA (RNCR)-2, long intergenic non-protein coding RNA p53 induced transcript (LINC-PINT)	[82,83,84]
	DNA methylation—Global DNA, 5,10-methylenetetrahydrofolate reductase (MTHFR) gene-promoter, miR-9-3 gene promoter	[81]

RNA, ribonucleic acid; DNA, deoxyribonucleic acid.

## Data Availability

Not applicable.
